# A New Midwives Bill

**Published:** 1899-01-07

**Authors:** 


### The Hospital

A Journal of

The Medical Sciences and Hospital Administration.

Vol. XXV.—No. 641.] Edited by Sir Henry Burdett, K.C.B., and Solomon C. Smith, M.D., M.R.C.P. [Jan. 7, 1899.

### A New Midwives Bill.

We have received from the Midwives Bill Committee a copy of their new Bill, which is stated to have been drafted on the lines of that introduced last year, but with certain alterations which have been made "in view of the requirements of the General Medical Council." Thus it is stated that: (1) Licensing is substituted for registration. (2) The habitual practice of midwifery for gain by women not licensed under this Bill is rendered illegal. (3) The area within which a midwife may practise is, in order to facilitate supervision, limited to that of the local supervising authority, from whom she has obtained a certificate authorising her to practise. Under the head of definitions, we find that "the term 'midwife' means a woman who undertakes for gain to attend women in child-birth." This is clearly an improvement upon that in the Bill of last year, in which the term was defined as "a woman who undertakes to attend cases of labour in accordance with the regulations to be laid down under this Act," thus leaving out the very women the control of whom is the one real excuse for legislation on the subject. Clause 2 (section 1) is strengthened in that, according to it, no woman is entitled to use the title of midwife, or any description implying that she is "qualified" to act as a midwife, unless she is licensed under the Act. This is an improvement, as in the old Bill the phrase used was "specially qualified." The whole essence of the Bill, however, so far as it concerns the outside world, and so far as it is to be effective in fulfilling its ostensible purpose and providing a better class of midwives for the poor, lies in clause 3 (section 2), which is as follows: "Any woman who, after the first day of January, one thousand nine hundred, not being licensed under this Act, shall take or use the name of midwife or any other such name, title, addition, or description as aforesaid, or shall habitually and for gain attend women in child-birth, shall be liable on summary conviction to a fine not exceeding five pounds." This is similar to the corresponding clause in the last Bill proposed by the British Medical Association, and it is this regulation of practice as well as title which makes the Bill a reality; and we hope that whatever may happen to the rest of the measure this clause will be retained in its entirety.

So far, we have nothing to say against the Draft Bill as submitted to us. We think, however, that there are some points further on which will tempt adverse criticism. Clause 4 provides that "Any woman who, within two years of the passing of this Act, claims to be licensed under this Act, shall be so licensed provided she holds a certificate in midwifery from the Royal College of Physicians of Ireland, or from the Obstetrical Society of London, or such other certificate as may be approved by the Central Midwives Board, or produces evidence as to character, and that she was in *bonâ fide* practice as a midwife at the passing of the Act." Thus, whatever the proposed Midwives Board may do as to others, the holders of the certificates issued by the two above-mentioned bodies are safe, and can demand their licences, character or no character. One wonders why? especially as this is entirely contrary to the recommendation of the Committee of the General Medical Council.

The authors of this Bill claim that "in view of the requirements of the General Medical Council, licensing is substituted for registration," and so far as the wording goes, it seems to be the case that the required verbal alterations have been made. But a careful reading of the Bill leads us to expect that the criticism will immediately be raised that no frank and open acceptance of the condition laid down by the Council is to be found in it. Truly, the term "Midwives' Register," which "means a register of midwives kept in pursuance of this Act," has vanished. But in its place we find "Midwives' Roll," which "means a roll of midwives kept in pursuance of this Act." Is this honest? Then the report of the General Medical Council said that the licence should "be granted by the local sanitary authority of the district" where the midwife practises. But the framers of this Bill, in their anxiety to get a register, or a "roll" as they now call it, make the Central Midwives Board "publish annually a roll of midwives who have been duly licensed under this Act," and make this Board "decide upon the removal from the roll of the name" of any offending midwife, "and also decide upon the restoration to the roll of the name of any midwife so removed," and also make the central body "issue licences." Surely, this "roll" is a "register," and nothing else. So far from complying with the "requirements" of the General Medical Council as to the licences being issued by the local sanitary authority, this Bill ordains that the "local supervising authority" (*i.e.*, the agent of the sanitary authority for purposes of the Act) shall have nothing to do with licensing the midwife, but shall merely "grant a certificate enabling her to practise" in his district, "which certificate *shall* be granted on proof by the woman that her name is on the midwives roll." There are many other points to which we might draw attention as showing that whatever else the Bill does, it does not carry out the recommendations of the General Medical Council.

We do not at present express any opinion upon this Bill as a whole, although we think that in some respects it is a distinct improvement on its predecessor. That it will be criticised, however, and criticised freely, we have no doubt, and among the first things upon which its enemies will fall will be the discrepancy between the "Memorandum" and the "Bill." Its authors must expect hard words.

